# Computational pathology in ovarian cancer

**DOI:** 10.3389/fonc.2022.924945

**Published:** 2022-07-29

**Authors:** Sandra Orsulic, Joshi John, Ann E. Walts, Arkadiusz Gertych

**Affiliations:** ^1^ Veterans Affairs Greater Los Angeles Healthcare System, Los Angeles, CA, United States; ^2^ Department of Obstetrics and Gynecology, David Geffen School of Medicine, University of California Los Angeles, Los Angeles, CA, United States; ^3^ Jonsson Comprehensive Cancer Center, University of California Los Angeles, Los Angeles, CA, United States; ^4^ Department of Psychiatry, David Geffen School of Medicine, University of California Los Angeles, Los Angeles, CA, United States; ^5^ Department of Pathology and Laboratory Medicine, Cedars-Sinai Medical Center, Los Angeles, CA, United States; ^6^ Department of Surgery, Cedars-Sinai Medical Center, Los Angeles, CA, United States; ^7^ Faculty of Biomedical Engineering, Silesian University of Technology, Zabrze, Poland

**Keywords:** artificial intelligence, computational pathology, convolutional neural network (CNN), deep learning, artificial neural network, digital pathology, machine learning, ovarian cancer

## Abstract

Histopathologic evaluations of tissue sections are key to diagnosing and managing ovarian cancer. Pathologists empirically assess and integrate visual information, such as cellular density, nuclear atypia, mitotic figures, architectural growth patterns, and higher-order patterns, to determine the tumor type and grade, which guides oncologists in selecting appropriate treatment options. Latent data embedded in pathology slides can be extracted using computational imaging. Computers can analyze digital slide images to simultaneously quantify thousands of features, some of which are visible with a manual microscope, such as nuclear size and shape, while others, such as entropy, eccentricity, and fractal dimensions, are quantitatively beyond the grasp of the human mind. Applications of artificial intelligence and machine learning tools to interpret digital image data provide new opportunities to explore and quantify the spatial organization of tissues, cells, and subcellular structures. In comparison to genomic, epigenomic, transcriptomic, and proteomic patterns, morphologic and spatial patterns are expected to be more informative as quantitative biomarkers of complex and dynamic tumor biology. As computational pathology is not limited to visual data, nuanced subvisual alterations that occur in the seemingly “normal” pre-cancer microenvironment could facilitate research in early cancer detection and prevention. Currently, efforts to maximize the utility of computational pathology are focused on integrating image data with other -omics platforms that lack spatial information, thereby providing a new way to relate the molecular, spatial, and microenvironmental characteristics of cancer. Despite a dire need for improvements in ovarian cancer prevention, early detection, and treatment, the ovarian cancer field has lagged behind other cancers in the application of computational pathology. The intent of this review is to encourage ovarian cancer research teams to apply existing and/or develop additional tools in computational pathology for ovarian cancer and actively contribute to advancing this important field.

## Computational imaging as a tool to study cancer from a novel perspective

Opinions about the role of computational imaging in the future of pathology range from the belief that computers will entirely replace pathologists to the conviction that computers will never achieve the competency of a well-trained pathologist. Similar to advanced chess-playing software, which is now practically unbeatable by humans, it is likely that computers will be faster and more accurate than pathologists in performing specific tasks, but the main purpose of computational pathology is not to outperform pathologists in routine analyses but to provide them with a completely new and complementary set of tools, some of which are highlighted below:

### Large-scale searchable integrative quantitation

Ovarian cancer is the fifth most deadly cancer in women, accounting for more than 200,000 deaths per year worldwide (1). Pathologic diagnosis is primarily confirmed by histopathologic evaluation and interpretation of H&E stained sections of tumor obtained during surgical debulking and/or biopsies obtained during treatment. Years of training with meticulous attention to histopathologic details in numerous examples of normal, altered but benign, and cancerous tissues enable pathologists to extract and empirically integrate an array of visual information from H&E slides into discrete features, such as tumor type, grade, mitotic count, and lymphovascular invasion. Using these features and additional pathological parameters allows the pathologist to categorize each tumor within a universally accepted classification, such as the World Health Organization (WHO) classification of tumors, which ultimately guides clinicians in patient management. Whereas each pathologist is variably limited by a composite of experience, memory, ability to recall visual detail, and case-correlated clinical outcome information locally available, computer algorithms take advantage of a searchable database with quantitative image features linked to clinical outcomes from thousands of patients. By applying computational image analyses, a new patient’s tumor can be compared to tumors in the database to find similar tumor phenotypes and help predict the most likely clinical outcome for the new patient.

### Subvisual features

**Figure 1:**
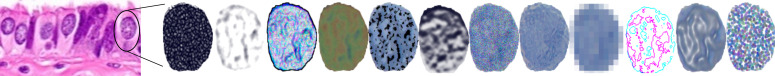
Artistic rendering of how a computer may “see” quantifiable nuclear features.

The invention of microscopes enabled the visualization of subcellular details, including nuclear enlargement, atypia, nucleolar prominence, hyperchromatism, and mitotic activity, which are usually important in establishing cancer diagnoses. However, a computer can extract and quantify hundreds, if not thousands, of additional visual and subvisual features (**Figure 1**), which can be configured into an algorithm to predict specific tumor behavior (i.e. likelihood of sensitivity to immunotherapy) or assess the efficacy of chemotherapy in tumor biopsies obtained at different time points during treatment. Extracting and analyzing information that is beyond human visual perception might also help identify new tissue, cellular, or subcellular structures, features, and/or spatial relationships that are difficult to characterize in H&E slides, such as liquid flow and vacuolarity, and intratumoral areas with increased tension, pH, metabolic activity, and/or genotoxic stress.

### Complex spatial information

**Figure 2:**
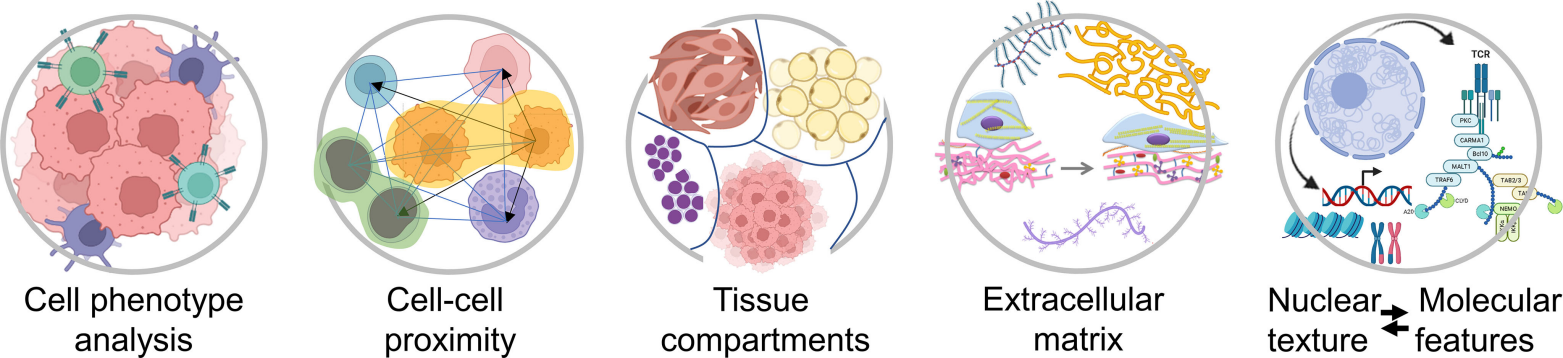
Examples of spatial characteristics within the tumor microenvironment that can be quantified using different types of computational image software. Created with BioRender.com.

In most solid malignancies, including ovarian cancer, cancer cells exist within a complex microenvironment that supports tumor growth. In general, the presence of immune cells is associated with better prognosis while the presence of cancer-associated fibroblasts (CAFs) is associated with worse prognosis. In addition to phenotypic diversity, it is becoming apparent that spatial relationships between cancer cells, immune cells, and CAFs play key roles in tumor aggressiveness and response to therapy. However, our knowledge and understanding about the composition of, and the spatial organization within, the tumor microenvironment is still in its infancy. Higher-order spatial patterns, such as cell-cell interactions, tissue interfaces, collagen alignment, and extent of tumor heterogeneity (**Figure 2**), are important readouts of the complexities of tumor pathophysiology and response to treatment yet remain largely unexplored. While the quantitation of individual cell types and the algorithms that are currently applied to summarize the extent and intensity of tumor staining in a biomarker panel are sufficient for patient stratification into broad groups, i.e. responders and non-responders, comprehensive spatial and morphometric readouts will be necessary to effectively tailor medical treatment to the individual characteristics of each patient (2, 3).

### Unbiased discovery

Knowing where and what to look for when examining a specimen or a histologic slide is crucial. Information that is not considered clinically relevant is more likely to be only cursorily examined or implicitly overlooked. For example, precursor lesions in the fallopian tube became obvious to pathologists only at the beginning of the 21^st^ century after decades of searching for these lesions in the ovary, which at the time seemed to be the most logical place for ovarian cancer initiation. Computers can record information extracted from hundreds of scanned slide images in a matter of hours. Using unbiased systematic processing of the recorded data, new and/or non-intuitive patterns can emerge (4). For example, although precursor lesions, such as serous tubal intraepithelial carcinoma (STIC) are identifiable in serially sectioned fallopian tubes processed with standard histopathology methods (5), the morphologic events that precede STIC formation are still unknown. Indeed, it is likely that STIC lesions are preceded by subtle morphologic changes that are either not visible to the human eye or are too nuanced to detect without prior knowledge of where and what to look for. A recent analysis of global stromal features in fallopian tubes from postmenopausal women showed that fallopian tubes with STIC lesions exhibit subvisual global changes in the stroma that might precede STIC formation and/or provide a permissible microenvironment for the neoplastic transformation of tubal epithelial cells (6). The ability to detect and understand what constitutes a “permissible” microenvironment for cancerous transformation provides new opportunities for the early detection of cancer and for the identification of rate-limiting events in the early stages of cancer development.

## Artificial intelligence, machine learning, and neural networks

Artificial Intelligence (AI) comprises a set of algorithms that mimic human intelligence, enabling machines to perform complex tasks, such as cognitive perception, decision-making, and communication. Machine learning is a subcategory of AI in which a machine is initially provided with large amounts of training data needed to build models to accurately analyze and interpret new data (7, 8). Machine learning models can automatically learn, improve their performance, and solve problems. Support vector machines, clustering algorithms, k-nearest neighbor classifiers, and logistic regression models (7, 8) are examples of commonly used models. However, because they can learn only a handful of features, they are being replaced by models that use deep learning. Deep learning is a type of machine learning that uses a neural network consisting of three or more functional layers, which include an input layer, multiple hidden layers, and an output layer (9, 10). The hidden layers promote learning and refinement of information “seen” by the input layers to increase the accuracy of predictions of the model’s output. The layers are connected to each other, and the strengths of these connections (termed “weights”) are learned from the training data.

A convolutional neural network (CNN) is a type of a deep learning model used to build advanced decision-making workflows in digital image analysis, ranging from image processing, classification and segmentation of tissue images to the prediction of patient outcomes. CNN often employs more than three convolutional sets of learnable filters (kernels) to extract features from the images. Kernel filters provide low, high and selective filtering (smoothing and sharpening, respectively). The filters distill various basic features such as edges, shapes, color, intensity and location of objects in the image. By flattening an image, removing or reducing the dimensions, convolutional kernels conduct pre-processing steps that enable selection and organization of the most informative learned features and recognition of these features in previously unseen images. In deeper layers of the CNN, the features are transformed into feature maps that summarize the presence of detected features in the input image (11). Another model that could prove useful in pathology is the fully convolutional network (FCN), characterized by a hierarchy of convolutional layers that are not fully connected. Unlike CNN, which learns from repetitive features that occur throughout the entire image, FCN learns from every pixel thereby allowing detection of objects or features that might be only sporadically present in an entire image (12), for example cancer stem cells which comprise a minute fraction of the tumor. CNN and FCN models are well suited for tumor/object detection, classification, and segmentation tasks (13–16). A recurrent neural network (RNN) model is characterized by dynamic behavior, meaning that it can memorize inputs over different time points or time intervals and learn from them in a sequential manner. RNN could be useful to analyze serial biopsies from a patient who is under surveillance for disease development, progression, or emergence of therapy resistance (17). Machine learning can be applied in a supervised or unsupervised manner. Supervised machine learning uses labeled data sets to train algorithms that classify data or predict outcomes. As input data are fed into the model, the significance of the individual data is adjusted until the model has been appropriately fitted to infer a function that maps input images to a designated outcome label or an object. In contrast, unsupervised machine learning uses unlabeled images to infer a function and detect patterns in the data, clustering them by any distinguishing characteristic(s). Both supervised and unsupervised learning can be useful to reveal new non-intuitive patterns that correlate with specific clinical outcomes.

## Computational pathology as a window into the molecular composition of cancer

Oncologists in consultation with pathologists increasingly rely on molecular assays to guide patient-tailored cancer therapy. Such assays can be costly and time-consuming in comparison to routine H&E slides, which can be digitized and analyzed on site. If the application of AI to H&E stained slides could provide molecular information, i.e. predict mutation status or expression levels of actionable cancer drivers in a patient’s tumor, several of the currently used technologies, such as immunohistochemistry, *in situ* hybridization, and next generation sequencing, might become superfluous. Recent efforts to integrate digitized H&E image data with molecular data indicate that determining molecular information from digitized H&E slides is not only feasible (18–21) but might achieve greater accuracy than other techniques that are subject to human error-prone steps, such as specialized specimen preparation and complex laboratory tasks.

Since tissue and cellular morphologies are known to reflect their physiological and pathological conditions, it would be reasonable to expect that higher-order features extracted from histopathology slides are associated with distinct molecular profiles, such as specific gene and protein expression, metabolism, and sensitivity to specific drugs. For example, a higher-order chromatin structure was shown to be the main determinant of genomic instability and mutation frequency in cancer cells (22–24) as well as a strong prognostic indicator in a pan-cancer study (25). Relying only on tumor histomorphology in H&E slides, recent reports indicate that it was possible to predict microsatellite instability in gastrointestinal cancers (26–28), determine molecular expression of ER, PR, and HER2 in breast cancer (29), and detect mutations in prognostically and therapeutically relevant driver genes in different cancer types, including BRAF in melanoma (30), APC, KRAS, PIK3CA, SMAD4, and TP53 in colorectal cancer (31), EGFR, KRAS, TP53, STK11, FAT1, and SETBP1 in lung cancer (32), TP53, CTNNB, FMN2, and ZFX4 in hepatocellular carcinoma (33), FGFR3 in bladder cancer (34), and IDH1 in glioma (35).

## Biomarker discovery

With an increasing number of available cancer therapies, it is crucial to develop companion diagnostic tests that identify and stratify subpopulations of patients by their likelihood to benefit from specific therapies. For example, in ovarian cancer, immunotherapy can be associated with serious side effects but only shows efficacy in about 10% of ovarian cancer patients (36). Another example is the need to identify patients who could benefit from PARP inhibitors. In the absence of better biomarkers, detection of a gene mutation (i.e. BRCA1 mutation) or elevated protein expression (i.e. PD-1 and PD-L1) of the intended therapeutic target are frequently used as proxies for treatment response. However, most of the current treatment response biomarkers perform poorly. While it is logical to expect that BRCA mutation carriers would be more sensitive to PARP inhibitors, clinical trials have shown that many non-carriers are responsive to PARP inhibitors although the rationale for this response is still unclear (37). Similarly, immunohistochemical detection of tumor-infiltrating lymphocytes and PD-L1 is a poor proxy for sensitivity to immunotherapy in most cancer types, including ovarian cancer (38). In fact, microsatellite instability, a less specific biomarker, seems to be a better predictor of sensitivity to immunotherapy in various solid cancers (39), possibly because microsatellite instability encompasses multiple proteins and pathways that are involved in the immune response. Using the same reasoning, it is likely that morphometric tumor features that reflect complex pathophysiological states will prove to be more clinically useful biomarkers even if they are initially non-intuitive or currently non-explainable.

Finding a biomarker in an unexpected place is exemplified in the tumor microenvironment. While cancer research has mostly focused on cancer cells, the unbiased application of deep imaging to digitized H&E slides combined with software that identifies areas of the image that influence algorithm-based classification has frequently revealed stromal features as better biomarkers of clinical outcomes than features of cancer cells (40). In ovarian cancer, a high ratio of stromal CAFs to cancer cells is an independent biomarker of poor survival and chemotherapy resistance (41–44). Indeed, stromal features were identified as the most effective biomarkers in colon cancer (45–47), mesothelioma (48), breast cancer (49, 50), lung adenocarcinoma (51) and early-stage non-small cell lung cancer (52). The discovery of stromal features as the most relevant predictors of clinical outcomes is not surprising as multiple molecular studies of expression profiles and proteomic signatures have also shown that an increased stroma/cancer ratio and increased expression of gene signatures associated with stromal remodeling were the strongest predictors of clinical outcomes (53–62). Studies in several cancer types have also shown that the signature of TGFβ-mediated extracellular matrix remodeling is the best predictor of therapy failure (63). The tumor stroma could contribute to poor survival and therapeutic resistance through multiple mechanisms, including promotion of tumor growth, angiogenesis, invasion and metastasis, provision of protective niches for cancer stem cells, creation of an immunosuppressive microenvironment that excludes immune cells from proximity to cancer cells, and/or generation of physical barriers that block access of chemotherapies and immunotherapies to cancer cells (64–68). Thus, it is unlikely that a single molecular pathway will be sufficient as a biomarker of the complex biology that dictates clinical outcomes. A recent computational image analysis of H&E slides of non-small cell lung and gynecologic cancers has shown that the spatial architecture and the interaction of cancer cells and tumor-infiltrating lymphocytes can predict clinical benefit in patients receiving immune checkpoint inhibitors. Importantly, the computational image classifier was associated with clinical outcome independent of clinical factors and PD-L1 expression levels (69). In addition to identifying new biomarkers by highlighting specific structures and regions, computational tissue imaging could assist with the identification of new therapeutic targets. For example, targeting CAFs with various TGFβ inhibitors was effective in improving immunotherapeutic efficacy in several preclinical cancer models (66–68).

## Future implementation of computational pathology in clinical practice

Potential applications of computational pathology in cancer include diagnosis, phenotyping, subtype classification, early detection, prognostication, assessment of sensitivity to chemotherapy and immunotherapy, and identification of suitable targeted therapies (20). For example, open-source software, QuPath (70), was capable of classifying serous borderline ovarian tumors and high-grade serous ovarian cancer with >90% accuracy by examining only a small number of tiles extracted from whole H&E slide images (71). While this approach does not reach the accuracy achieved by pathologists who examine multiple sections from different areas of each tumor and have access to surgical records and other clinical information, this example suggests that improvements in the software recognition pattern and computational capacity to analyze pathology slides could achieve clinical-grade tumor classification (72).

Several examples report the utility of computational imaging to automate cancer diagnoses without compromising accuracy. For example, a CNN trained to classify images as malignant melanoma or benign nevi demonstrated superior performance compared to manual scoring by histopathologists (73, 74). Another study that assessed the ability of deep learning algorithms to accurately detect breast cancer metastases in H&E slides of lymph node sections reported that the algorithms were superior in detecting micrometastases and equivalent to the best performing pathologists when under time constraints in detecting macrometastases (75). Similarly, an AI system has reached a clinically acceptable level of cancer detection accuracy in prostate needle biopsies (76). A study of deep CNN in Gleason scoring of prostatectomy specimens reported that the deep learning-based Gleason classification achieved higher sensitivity and specificity than 9 out of 10 pathologists (77). Computational analyses of H&E images have been applied for the prediction of patient survival and recurrence. Deep learning-based algorithms have also been reported to predict prostate cancer recurrence comparably to a genomic companion diagnostic test (78). Other studies utilizing deep learning-based classification involve determining histologic subtype in ovarian cancer (79), predicting platinum resistance in ovarian cancer (41, 80), predicting survival outcome in patients with mesothelioma (48), predicting metastatic recurrence and death in patients with primary melanoma (81) and colon cancer (46), predicting lung cancer recurrence after surgical resection to identify patients who should receive additional adjuvant therapy (52, 82), and predicting response to ipilimumab immunotherapy in patients with malignant melanoma (83). A computational quantitative characterization of the architecture of tumor-infiltrating lymphocytes and their interplay with cancer cells from H&E slides of three different gynecologic cancer types (ovarian, cervical, and endometrial) and across three different treatment approaches (platinum, radiation and immunotherapy) showed that the geospatial profile was prognostic of disease progression and survival irrespective of the treatment modality (84). This computationally-derived profile even outperformed the stage variable, which is the current standard for gynecologic oncology (84). Upon closer inspection, features related to the increased density of lymphocytes in the epithelium and invasive tumor front were associated with better survival compared with features related to the increased density of lymphocytes in the stromal compartment (84). Although these patterns can be observed by conventional pathology examination, the value of the computational geospatial profile is in the inherently quantitative output rather than the descriptive output from conventional pathology.

Computational pathology has already become an integral component of cancer research and is increasingly being used in anatomic pathology to automate tasks, reduce subjectivity, and improve accuracy and reproducibility (17, 85, 86). The development of high-resolution whole slide image scanners, which are specialized microscopes fitted with high resolution cameras, high magnification objectives, and software for the identification and automated correction of out-of-focus points, constitutes a major advance in computational pathology. Two of these high-quality scanners, the Leica Aperio AT2 DX System and the Philips IntelliSite Pathology Solution, now have FDA approval for clinical review and expert interpretation of pathology slides in routine diagnoses (87). To facilitate the adoption and implementation of this technology into pathology workflows, the Digital Pathology Association has published several practical guides to whole slide imaging (88–91). Digitization of pathology slides enables simultaneous review of slides by multiple pathologists at different institutions and the creation and growth of centralized cloud-based image repositories and databases that are accessible for remote high-throughput studies and/or the development of multiple freely-available machine learning-based tools that can recognize and quantify positive immunostaining or basic structures, such as epithelial-lined ducts, blood vessels, and mitotic figures in H&E slides. Proof-of-concept studies show that automated analyses of simple morphologic patterns can assist pathologists and researchers, allowing them to focus on more complex tasks (2, 92).

Academic pathology departments are increasingly interested in adopting AI in routine practice to optimize operations, reduce costs, and improve patient care. A survey of physician perspectives on the integration of AI into diagnostic pathology revealed that 75% of pathologists across more than fifty countries are interested in using AI as a diagnostic tool in cancer care (93). With the continuous increase in the aged population and the growing number of available tests for persionalized medicine, pathologists are overburdened by tasks, such as counting mitotic figures or scoring Ki67 staining, that could be easily performed by machines. Hence, automated computational imaging platforms for the detection and quantitation of stained markers in defined cell subpopulations are the first candidates to be integrated into pathology workflows as these analyses could provide accurate, unbiased, reproducible, and standardized results that can be viewed and verified (72, 89, 94–96). In contrast, results of artificial neural network analyses that cannot be verified visually are more likely to be met with mistrust by the pathology community because the pathologist needs to be confident in the result before signing out a report (97). This problem might be solved by the further development of tools that introduce transparency for non-linear machine learning methods, such as gradient-weighted class activation mapping (grad-CAM) that overlays images and heatmaps to better visualize the cell type or region in which the informative features were expressed (98).

Although research results utilizing computational imaging in pathology are promising, significant improvements need to be made to achieve the safety and reliability required for endorsement by medical specialty societies, legal approvals, and implementation of this technology into daily clinical pathological diagnostics (99–101). One of the first steps will involve the standardization of slide scanners, display devices, image formats, platforms for image processing and analysis as well as the standardization of the interfaces with clinical information systems and reimbursement mechanisms. Among the technical challenges that must be overcome are normalization methods for histological data preparation at different institutions. This is a pressing issue given that histologic slides are stained either manually or with auto-stainers using site-specific protocols and then digitized on scanners from various manufacturers. Tissue handling and processing can also introduce artefacts, such as air bubbles, fingerprints, excessive glue, blurry/out of focus tissue image, unstained or overstained tissue, and folded or cauterized tissue and digitization (102). While the human eye might not be sensitive to slight differences in tissue fixation, sectioning, staining, and coverslip mounting, all of these factors indiscriminately impact machine learning, and each can have drastic implications on the final results (102). For these reasons, the computational pathology community strongly promotes rigorous algorithm development and testing schemes. First, innovative methods must be used to address pixel data normalization, color standardization, and recognition of the areas on the slide that are out of focus, blurred or contain folded or damaged tissue (103–109). Second, algorithm generalizability on unseen digitized specimens collected at different institutions or deposited in public repositories, such as The Cancer Genome Atlas (TCGA) (110) and The Cancer Imaging Archive (TCIA) (111), will need to be tested and standardized (7, 112, 113).

Many commercial and freely available viewers and image analysis software packages support different whole slide image formats for Windows, Linux, and MacOS. Commercial slide viewers include ImageScope (Aperio/Leica), CaseViewer (3DHistech), and ObjectiveView. The development of web-based viewing interfaces and custom computational pathology pipelines is supported by OpenSlide and SlideIO software libraries. For users with little to no programming skills, the most popular free software packages for general tissue image analysis include ImageJ (114), Fiji (115), Icy (116), Orbit (117), ilastik (118), Cell Profiler (119), QuPath (70), SlicerScope (120), and PathML (121). These platforms have been embraced in the research arena because they can be easily adapted to different types of image analyses by introducing new codes or incorporating plugins from other programs. They are also flexible in processing different types of image files. Additionally, a plethora of fit-for-purpose packages with custom-made computational pathology pipelines can be found on GitHub (https://github.com/). Commercial digital pathology solutions, such as PerkinElmer (USA), Tissue Gnostics (Austria), Halo (United States), Visophram (Denmark) and Definiens (Germany), are often preferred by pathology departments because these platforms offer built-in databases of analytical engines and available technical support. Recognizing the importance of standardization in clinical practice, the freely-accessible Pathology Analytic Imaging Standards (PAIS) was created to support the standardization of image analysis algorithms and image features (122, 123). Resources of web-based interfaces and online tools, such as The Cancer Digital Slide Archive (CDSA, http://cancer.digitalslidearchive.net/), for visualization and analysis of pathology image data are also becoming more readily available (124).

## Conclusion

Computational image analysis holds great promise in advancing ovarian cancer research, including a better understanding of the events that provide a permissible microenvironment for early cancerous transformation in the fallopian tube, the role of the stroma in therapeutic resistance, and biomarkers for the selection of patients most likely to benefit from different therapeutic approaches. Although computational pathology in the ovarian cancer research field is still too rudimentary to use in a clinical setting, we anticipate that it will become one of the main tools for cancer research and precision medicine in the next decade, surpassing current methods for the selection of patient-tailored therapy, such as immunostaining, mutation sequencing, and expression profiling. Among the key factors that will propel computational pathology to the forefront of medical research are the accessibility of histopathology slides, the low-cost of image analysis and storage, seamless integration with other computational imaging platforms, and the potential for rapid, accurate, and reliable prediction of patient outcomes.

## Author contributions

All authors contributed to the article writing and revisions and approved the submitted version.

## Funding

SO is supported by the Veterans Administration Merit Award VA-ORD BX004974, the Iris Cantor-UCLA Women’s Health Center/UCLA National Center of Excellence in Women’s Health Pilot Research Project NCATS UCLA CTSI grant UL1TR00188, the Sandy Rollman Ovarian Cancer Foundation, the Mary Kay Foundation, and the Annenberg Foundation. AG is supported by the Initiative of Excellence - Research University - a grant program at Silesian University of Technology (year 2021, no.07/010/SDU/10-21-01).

## Conflict of interest

The authors declare that the research was conducted in the absence of any commercial or financial relationships that could be construed as a potential conflict of interest.

## Publisher’s note

All claims expressed in this article are solely those of the authors and do not necessarily represent those of their affiliated organizations, or those of the publisher, the editors and the reviewers. Any product that may be evaluated in this article, or claim that may be made by its manufacturer, is not guaranteed or endorsed by the publisher.
